# *In Vivo* Generation of BK and JC Polyomavirus Defective Viral Genomes in Human Urine Samples Associated with Higher Viral Loads

**DOI:** 10.1128/JVI.00250-21

**Published:** 2021-05-24

**Authors:** Amin Addetia, Quynh Phung, Benjamin T. Bradley, Michelle J. Lin, Haiying Zhu, Hong Xie, Meei-Li Huang, Alexander L. Greninger

**Affiliations:** a Department of Laboratory Medicine and Pathology, University of Washington, Seattle, Washington, USA; b Molecular and Cellular Biology Graduate Program, University of Washington, Seattle, Washington, USA; c Vaccine and Infectious Disease Division, Fred Hutchinson Cancer Research Center, Seattle, Washington, USA; International Centre for Genetic Engineering and Biotechnology

**Keywords:** BK virus, DNA virus, JC virus, ddPCR, defective, defective interfering particle, defective interfering genome, defective viral genome, polyomavirus, rearrangement

## Abstract

Defective viral genomes (DVGs) are parasitic viral sequences containing point mutations, deletions, or duplications that might interfere with replication. DVGs are often associated with viral passage at high multiplicities of infection in culture systems but have been increasingly reported in clinical specimens. To date however, only RNA viruses have been shown to contain DVGs in clinical specimens. Here, using direct deep sequencing with multiple library preparation strategies and confirmatory digital droplet PCR (ddPCR) of urine samples taken from immunosuppressed individuals, we show that clinical BK polyomavirus (BKPyV) and JC polyomavirus (JCPyV) strains contain widespread genomic rearrangements across multiple loci that likely interfere with viral replication. BKPyV DVGs were derived from BKPyV genotypes Ia, Ib-1, and Ic. The presence of DVGs was associated with specimens containing higher viral loads but never reached clonality, consistent with a model of parasitized replication. These DVGs persisted during clinical infection as evidenced in two separate pairs of samples containing BK virus collected from the same individual up to 302 days apart. In a separate individual, we observed the generation of DVGs after a 57.5-fold increase in viral load. In summary, by extending the presence of DVGs in clinical specimens to DNA viruses, we demonstrate the ubiquity of DVGs in clinical virology.

**IMPORTANCE** Defective viral genomes (DVGs) can have a significant impact on the production of infectious virus particles. DVGs have only been identified in cultured viruses passaged at high multiplicities of infection and RNA viruses collected from clinical specimens; no DNA virus in the wild has been shown to contain DVGs. Here, we identified BK and JC polyomavirus DVGs in clinical urine specimens and demonstrated that these DVGs are more frequently identified in samples with higher viral loads. The strains containing DVGs had rearrangements throughout their genomes, with the majority affecting genes required for viral replication. Longitudinal analysis showed that these DVGs can persist during an infection but do not reach clonality within the chronically infected host. Our identification of polyomavirus DVGs suggests that these parasitic sequences exist across the many classes of viruses capable of causing human disease.

## INTRODUCTION

Defective viral genomes (DVGs) constitute a peculiar group of viral mutants incapable of autonomous replication. These DVGs contain point mutations, deletions, or duplications in their genomes (1). DVGs can either hinder or assist the growth of the virus. The plant pathogen *Turnip crinkle virus* produces DVGs that assist viral growth by interfering with the plant’s antiviral mechanisms (2, 3). Many DVGs of human-pathogenic viruses produce defective interfering particles that parasitize and interfere with viral replication. These DVGs are thought to contribute to chronic viral infections by promoting survival of the infected host cells or reducing the infectious viral load by suppressing the number of replication-competent virions produced during active infection (4). Increasingly, DVGs are being investigated for their potential as antiviral strategies (1, 5).

Two major types of DVGs have been described for RNA viruses (1). Deletion-type DVGs are truncated versions of the nondefective viral genome that occur when the polymerase skips part of the genome during replication. Deletion DVGs tend to possess identical terminal sequences while missing several or all essential genes required for self-propagation (6, 7). The second type of DVG is the copy-back DVGs. Copy-back and the related snap-back DVGs are rearranged genomes consisting of an authentic terminus followed immediately by an inverted repeat of some or all of that sequence (8). Copy-back DVGs have been reported in many negative-sense RNA viruses, mainly in the *Paramyxoviridae* family, and are predicted to be the products from the reattachment of RNA-dependent RNA polymerase (RdRp) to the nascent strand, thus copying back the end of the genome (9).

DVGs have frequently been identified in cell culture systems, especially when viral stocks are passaged at high multiplicities of infection, and are increasingly being identified in clinical specimens (4, 10–14). These DVGs contain deletions of variable lengths and across various regions in the genome but generally retain the origin of replication (15). Cultured BK and JC polyomavirus have both been shown to contain deletions spanning the large T antigen (16, 17). Deletions spanning multiple genomic regions, including VP1 and VP2, have been observed when the related simian virus 40 is passaged in cell culture (18–21). To date, only single-stranded RNA viruses have been demonstrated to form DVGs in clinical specimens (4, 22–24). However, the significance and role of DVG during clinical infection are unclear.

Polyomaviruses are nonenveloped, double-stranded DNA viruses with a small, circular genome of approximately 5,000 bp (25). Among the 102 identified species, BK polyomavirus (BKPyV) and JC polyomavirus (JCPyV) are the two most commonly known to infect humans (26). BKPyV and JCPyV are highly prevalent in the population, with most individuals initially getting infected in early childhood and maintaining a lifelong infection thereafter (27, 28). The infections of BKPyV, also known as *Human polyomavirus 1*, are not associated with disease in immunocompetent individuals but can cause nephropathy and allograft failure in individuals receiving a renal transplant (29–32). In individuals with hemorrhagic cystitis, BKPyV can be found at very high titers in urine (>10^8^ copies/ml of urine) (10). JCPyV, also known as *Human polyomavirus 2*, is most well known for its association with progressive multifocal leukoencephalopathy (PML) in immunosuppressed individuals and can cause many novel neurological disorders, such as JC virus granule cell neuropathy and JC virus encephalopathy (11). JCPyV can be found in cerebrospinal fluid and urine of individuals with PML at values ranging from 10^2^ to 10^8^ copies/ml (12).

While performing genome recovery of polyomaviruses for urine specimens sent to our clinical laboratory, we noticed many large-scale genomic rearrangements in BKPyV and JCPyV. These rearrangements were recovered independently of the sequencing strategy used and were found across different viral lineages. Polyomavirus DVGs were found more frequently in those specimens that were specifically associated with high viral load specimens but never reached clonality, consistent with a model of defective interfering replication.

## RESULTS

### Defective polyomavirus genomes in clinical urine samples.

**FIG 1:**
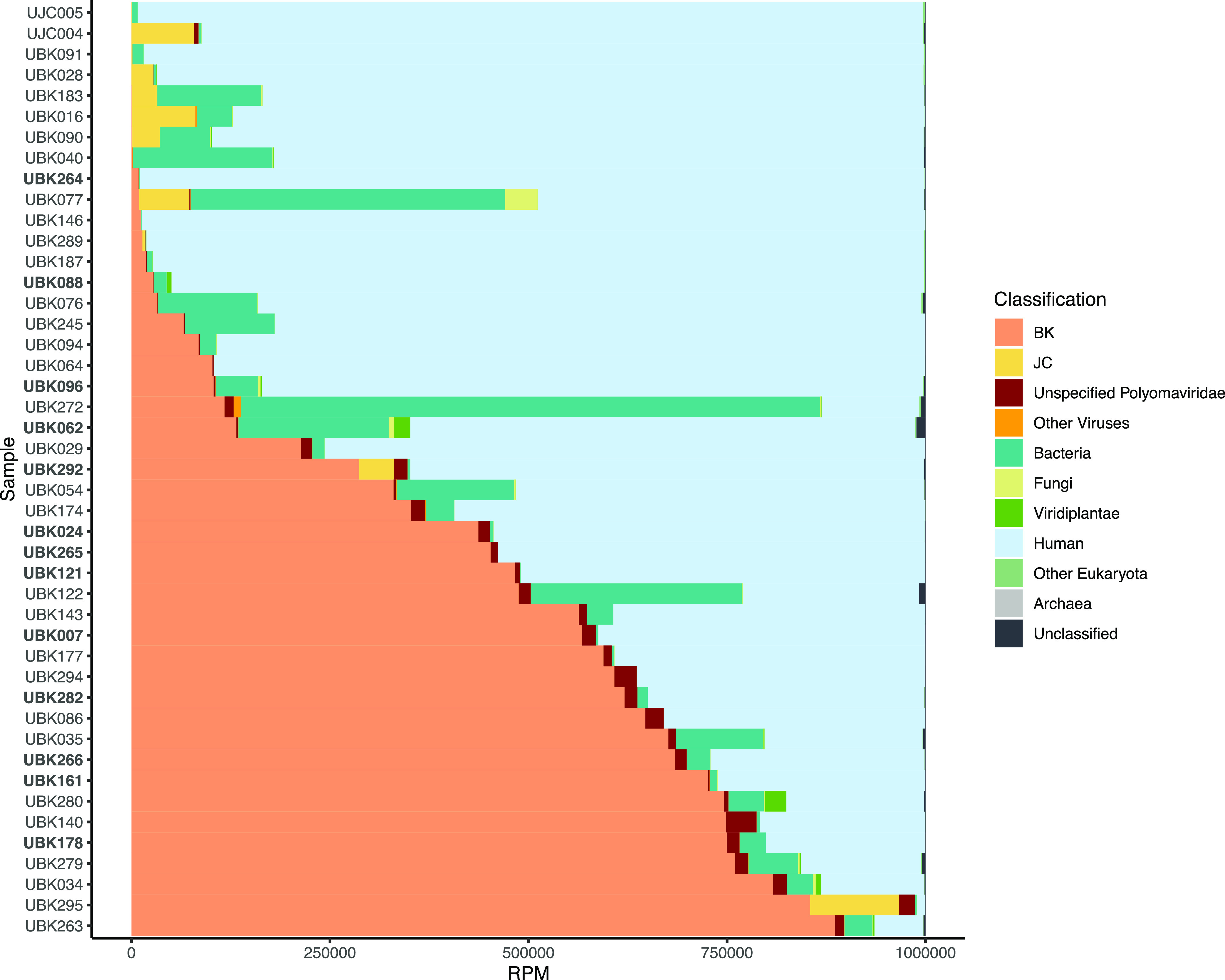
Taxonomic classifications identified by metagenomic analysis, color-coded and normalized by reads per million (RPM). Samples are sorted in ascending order of RPM assigned to BKPyV. The samples with bolded labels contain DVGs.

We performed metagenomic shotgun sequencing on DNA extracted from 43 BKPyV-positive clinical urine samples collected from 37 individuals with a median viral load of 1.50 × 10^8^ copies/ml (range, 6.3 × 10^4^ to 4.6 × 10^10^ copies/ml) and 2 JCPyV-positive clinical urine specimens (see Table S1 in the supplemental material). Of the 45 samples, we recovered 38 complete BKPyV genomes and 11 complete JCPyV genomes and found BKPyV and JCPyV coinfections in 11 of these samples (Fig. 1). We identified cooccurrence of common uropathogenic bacteria present at a frequency greater than 10 reads per million (RPM) in 30 of the 45 polyomavirus-positive samples (Table S2). Reads corresponding to *Candida* spp. at a frequency greater than 10 RPM were identified in 9 of the 45 samples.

**FIG 2:**
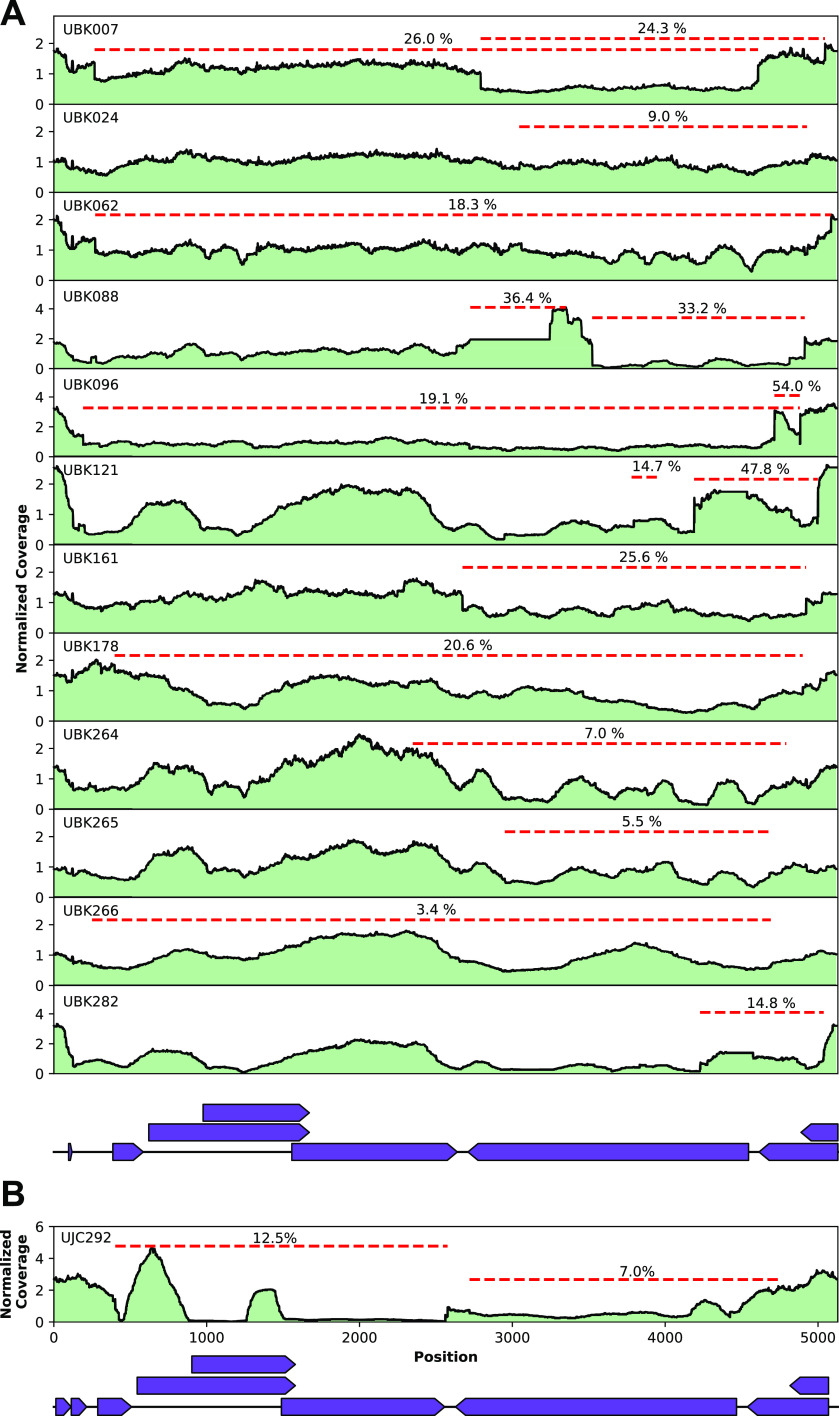
(A and B) Coverage maps of defective BKPyV genomes (A) and a JCPyV genome (B) observed in shotgun sequencing data of 13 polyomavirus-positive specimens. The normalized sequencing read coverage across each sample’s consensus viral genome sequence is plotted in green. Junctions present in individual sequencing reads that are representative of the genomic rearrangements present in the DVGs are depicted by red dashes. For these rearrangements, the percentage of reads with the particular junction was calculated relative to the maximum genomic coverage in the analyzed sample.

In 13 of the BKPyV-positive samples, we identified large rearrangements or deletions constituting DVGs in BKPyV or JCPyV that were supported by 10 or more sequencing reads and included samples with a sum total frequency of 10% or greater of these rearrangements (Fig. 2, Table 1). Twelve of the thirteen samples with rearrangements were identified in BKPyV genomes collected from eleven different individuals, while the remaining sample was identified in a JCPyV genome.

**TABLE 1:** Rearrangement and deletion junction positions and counts for BKPyV DVGs as detected by Geneious Prime using a threshold of at least 10 reads and frequency >10% compared to maximum depth

Sample	Junction type	Nucleotide start position	Nucleotide end position	Length (bp)	No. of Junction reads	Reads with junction (%)	Genes affected
UBK007	Rearrangement	268	4628	763	958	26.0	LargeT, SmallT
	Rearrangement	2805	5068	2,262	898	24.3	LargeT, SmallT
	Deletion	236	346	110	312	8.5	None
	Deletion	260	346	86	94	2.6	None
	Deletion	275	428	153	67	1.8	Agnoprotein
	Deletion	317	324	7	45	1.2	None
	Rearrangement	262	4794	591	26	0.7	Agnoprotein, LargeT, SmallT
	Deletion	2914	3826	912	25	0.7	LargeT
UBK024	Deletion	1739	1746	7	546	25.0	VP1
	Deletion	4841	4971	130	320	14.6	LargeT, SmallT
	Rearrangement (inversion)	3059	4950	1,891	196	9.0	LargeT, SmallT
	Rearrangement (inversion)	61	3155	2,045	148	6.8	Agnoprotein, LargeT, VP1, VP2, VP3
	Deletion	4647	4768	121	137	6.3	SmallT
	Rearrangement (inversion)	243	3354	2,028	97	4.4	Agnoprotein, LargeT, VP1, VP2, VP3
	Rearrangement	76	4567	649	95	4.3	Agnoprotein, LargeT, VP1, VP2, VP3
	Rearrangement (inversion)	47	306	259	78	3.6	None
	Rearrangement (inversion)	3060	4974	1,914	77	3.5	LargeT, SmallT
	Deletion	5119	218	510	72	3.3	LargeT, SmallT
	Rearrangement	611	405	207	51	2.3	All
	Rearrangement	5011	4981	31	43	2.0	All
	Rearrangement	165	2215	2,049	27	1.2	Agnoprotein, VP1, VP2, VP3
UBK062	Rearrangement	272	5108	292	2,696	18.3	All
	Rearrangement	49	2896	2,281	1,800	12.2	Agnoprotein, LargeT, VP1, VP2, VP3
	Rearrangement	3074	4953	1,878	1,123	7.6	LargeT, SmallT
	Deletion	2894	3272	378	1,009	6.8	LargeT
	Rearrangement	2920	4168	1,247	952	6.5	LargeT
	Rearrangement	3272	49	1,903	811	5.5	LargeT, SmallT
	Deletion	3461	4191	730	603	4.1	LargeT
	Rearrangement	220	4847	501	578	3.9	All
	Rearrangement	327	5019	436	423	2.9	All
	Rearrangement	3325	4981	1,655	239	1.6	LargeT, SmallT
	Rearrangement	2779	4051	1,271	200	1.4	LargeT
	Rearrangement	290	4951	467	152	1.0	All
	Rearrangement	288	2267	1,978	116	0.8	Agnoprotein, VP1, VP2, VP3
	Rearrangement	255	4925	458	111	0.8	All
	Rearrangement	303	4943	488	109	0.7	All
	Deletion	4183	4682	499	102	0.7	LargeT, SmallT
	Rearrangement	2784	4872	2,087	101	0.7	LargeT, SmallT
	Deletion	2904	3490	586	99	0.7	LargeT
	Rearrangement	480	2805	2,324	89	0.6	Agnoprotein, LargeT, VP1, VP2, VP3
	Rearrangement	3118	4872	1,753	87	0.6	LargeT, SmallT
	Rearrangement	3634	4741	1,106	67	0.5	LargeT, SmallT
	Rearrangement	273	4678	723	66	0.5	All
	Rearrangement	285	4856	557	64	0.4	All
	Rearrangement	330	4962	496	47	0.3	All
	Deletion	3382	4314	932	45	0.3	LargeT
	Deletion	3752	4673	921	40	0.3	LargeT, SmallT
	Deletion	696	700	4	37	0.3	VP2
	Deletion	4899	5098	199	36	0.2	LargeT, SmallT
	Rearrangement	360	4297	1,191	34	0.2	Agnoprotein, LargeT, VP1, VP2, VP3
	Rearrangement	3037	2850	188	18	0.1	All
	Rearrangement	271	3065	2,334	16	0.1	Agnoprotein, LargeT, VP1, VP2, VP3
	Deletion	307	692	385	15	0.1	Agnoprotein, VP2
	Rearrangement	231	5089	270	15	0.1	All
	Rearrangement	391	4634	885	13	0.1	Agnoprotein, LargeT, VP1, VP2, VP3
	Rearrangement (inversion)	1141	1179	38	13	0.1	VP2, VP3
	Deletion	391	1047	656	12	0.1	Agnoprotein, VP2, VP3
	Deletion	492	705	213	10	0.1	Agnoprotein, VP2
UBK088	Deletion	2737	3365	628	219	36.4	LargeT
	Rearrangement	3538	4935	1,396	200	33.2	LargeT, SmallT
	Rearrangement	277	4836	551	56	9.3	All
	Rearrangement	3379	245	1,974	51	8.5	LargeT, SmallT
	Rearrangement	3338	3272	67	27	4.5	All
UBK096	Rearrangement (inversion)	4736	4905	169	525	54.0	SmallT
	Rearrangement (inversion)	192	4903	430	186	19.1	All
	Rearrangement	2731	4963	2,231	68	7.0	LargeT, SmallT
	Rearrangement	300	4650	792	48	4.9	All
	Deletion	183	322	139	31	3.2	None
	Rearrangement (inversion)	3780	3825	45	23	2.4	LargeT
	Rearrangement	24	1851	1,826	19	2.0	Agnoprotein, VP1, VP2, VP3
	Rearrangement	2796	5043	2,246	14	1.4	LargeT, SmallT
	Rearrangement	4740	2337	2,404	12	1.2	All
UBK121	Deletion	4382	4558	176	6,030	55.7	LargeT
	Rearrangement (inversion)	4208	5021	813	5174	47.8	LargeT, SmallT
	Deletion	1898	1902	4	3,634	33.5	VP1
	Deletion	3747	3756	9	1,700	15.7	LargeT
	Deletion	3797	3984	187	1,587	14.7	LargeT
	Rearrangement	4847	3811	1,037	881	8.1	LargeT, SmallT
	Rearrangement (inversion)	4210	5017	807	774	7.1	LargeT, SmallT
	Deletion	4682	4855	173	753	7.0	SmallT
	Rearrangement (inversion)	96	4749	442	653	6.0	All
	Rearrangement	4302	2962	1,341	611	5.6	All
	Rearrangement (inversion)	4207	5020	813	583	5.4	LargeT, SmallT
	Deletion	3501	3511	10	486	4.5	LargeT
	Rearrangement	4104	3467	638	435	4.0	All
	Deletion	2843	3499	656	271	2.5	LargeT
	Rearrangement	3624	4989	1,364	246	2.3	LargeT, SmallT
	Deletion	1883	1890	7	196	1.8	VP1
	Rearrangement	3341	32	1,785	171	1.6	LargeT, SmallT
	Deletion	4587	4677	90	152	1.4	SmallT
	Rearrangement	3461	3,455	7	113	1.0	All
	Deletion	2678	2686	8	99	0.9	None
	Rearrangement (inversion)	3097	5,046	1,949	83	0.8	LargeT, SmallT
	Rearrangement (inversion)	4818	5070	252	82	0.8	LargeT, SmallT
	Rearrangement (inversion)	3635	4972	1,337	69	0.6	LargeT, SmallT
	Rearrangement (inversion)	4313	4817	504	63	0.6	LargeT, SmallT
	Rearrangement (inversion)	2770	4570	1,800	51	0.5	LargeT
	Rearrangement (inversion)	3109	4254	1,145	50	0.5	LargeT
	Deletion	4,641	4846	205	50	0.5	SmallT
	Deletion	4308	4778	470	42	0.4	LargeT, SmallT
	Rearrangement	80	4639	537	32	0.3	All
	Deletion	3612	3616	4	29	0.3	LargeT
	Deletion	3550	4185	635	28	0.3	LargeT
	Deletion	664	668	4	26	0.2	VP2
	Rearrangement (inversion)	155	4625	625	26	0.2	Agnoprotein, LargeT, VP1, VP2, VP3
	Rearrangement (inversion)	4694	4727	33	26	0.2	SmallT
	Rearrangement (inversion)	3153	3984	831	24	0.2	LargeT
	Deletion	4788	4794	6	22	0.2	SmallT
	Rearrangement (inversion)	3594	4299	705	21	0.2	LargeT
	Deletion	4,694	4704	10	19	0.2	SmallT
	Deletion	4888	5094	206	18	0.2	LargeT, SmallT
	Rearrangement (inversion)	24	4273	846	18	0.2	Agnoprotein, LargeT, VP1, VP2, VP3
UBK161	Rearrangement	2686	4944	2,257	1,369	25.6	LargeT, SmallT
	Rearrangement	501	2259	1,757	426	8.0	All
	Rearrangement	2246	5041	2,318	82	1.5	LargeT, SmallT, VP1
	Deletion	3476	3509	33	29	0.5	LargeT
	Rearrangement	472	1707	1,234	28	0.5	Agnoprotein, VP1, VP2, VP3
	Rearrangement (inversion)	320	340	20	20	0.4	None
	Deletion	681	685	4	14	0.3	VP2
	Rearrangement	5094	1970	1,987	14	0.3	All
UBK178	Rearrangement	400	4922	620	1,099	20.6	All
	Deletion	2931	3865	934	952	9.5	LargeT
	Rearrangement (inversion)	288	3478	1,951	601	6.0	Agnoprotein, LargeT, VP1, VP2, VP3
	Rearrangement (inversion)	4638	5037	399	590	5.9	LargeT, SmallT
	Deletion	5079	365	530	34	0.3	LargeT, SmallT
	Deletion	710	714	4	32	0.3	VP2
	Rearrangement (inversion)	2633	2711	78	31	0.3	VP1
	Rearrangement	353	293	61	27	0.3	All
	Rearrangement	2413	4899	2,485	22	0.2	LargeT, SmallT, VP1
	Rearrangement (inversion)	3199	3356	157	22	0.2	LargeT
	Rearrangement	478	1,534	1,055	19	0.2	Agnoprotein, VP2, VP3
	Deletion	2488	2,502	14	18	0.2	VP1
	Deletion	158	301	143	16	0.2	None
	Rearrangement (inversion)	1838	2150	312	16	0.2	VP1
	Rearrangement	66	4748	460	15	0.2	All
	Rearrangement (inversion)	1453	1463	10	14	0.1	VP2, VP3
	Rearrangement (inversion)	137	4957	321	14	0.1	All
	Rearrangement (inversion)	2808	3360	552	13	0.1	LargeT
	Deletion	3446	3458	12	11	0.1	LargeT
	Deletion	477	583	106	10	0.1	Agnoprotein
UBK264	Rearrangement	2359	4814	2,454	14	7.0	LargeT, SmallT, VP1
	Rearrangement	276	1892	1,615	12	6.0	Agnoprotein, VP1, VP2, VP3
UBK265	Rearrangement	2964	4702	1,737	553	5.5	LargeT, SmallT
	Rearrangement	203	2891	2,454	305	3.0	Agnoprotein, LargeT, VP1, VP2, VP3
	Rearrangement	304	2360	2,055	215	2.1	Agnoprotein, VP1, VP2, VP3
	Rearrangement	2622	4703	2,080	104	1.0	LargeT, SmallT, VP1
	Deletion	270	311	41	87	0.9	None
	Rearrangement	3827	4873	1,045	65	0.6	LargeT, SmallT
	Rearrangement	3429	4755	1,325	52	0.5	LargeT, SmallT
	Rearrangement (inversion)	3894	4519	625	50	0.5	LargeT
	Rearrangement (inversion)	275	4495	921	49	0.5	Agnoprotein, LargeT, VP1, VP2, VP3
	Deletion	710	714	4	49	0.5	VP2
	Deletion	180	354	174	49	0.5	None
	Rearrangement	282	2741	2,458	42	0.4	Agnoprotein, LargeT, VP1, VP2, VP3
	Rearrangement	230	3539	1,833	39	0.4	Agnoprotein, LargeT, VP1, VP2, VP3
	Rearrangement	3813	5001	1,187	37	0.4	LargeT, SmallT
	Rearrangement	218	3599	1,761	32	0.3	Agnoprotein, LargeT, VP1, VP2, VP3
	Deletion	3867	4649	782	32	0.3	LargeT, SmallT
	Deletion	3019	3925	906	30	0.3	LargeT
	Rearrangement	3733	5104	1,370	27	0.3	LargeT, SmallT
	Rearrangement	282	3837	1,587	26	0.3	Agnoprotein, LargeT, VP1, VP2, VP3
	Rearrangement	263	2914	2,491	26	0.3	Agnoprotein, LargeT, VP1, VP2, VP3
	Rearrangement	281	4554	869	26	0.3	Agnoprotein, LargeT, VP1, VP2, VP3
	Deletion	498	517	19	25	0.3	Agnoprotein
	Rearrangement	261	4826	577	25	0.3	All
	Deletion	252	349	97	25	0.3	None
	Rearrangement	263	4455	950	24	0.2	Agnoprotein, LargeT, VP1, VP2, VP3
	Rearrangement	227	4703	666	24	0.2	All
	Rearrangement	266	4703	705	24	0.2	All
	Rearrangement (inversion)	31	390	359	21	0.2	Agnoprotein
	Rearrangement	280	4771	651	19	0.2	All
	Rearrangement	3100	4491	1,390	15	0.2	LargeT
	Deletion	252	357	105	14	0.1	None
UBK266	Rearrangement	251	4712	681	83	3.4	All
	Rearrangement (inversion)	13	307	294	69	2.8	None
	Rearrangement	3643	4721	1,077	48	2.0	LargeT, SmallT
	Rearrangement	313	4610	845	42	1.7	Agnoprotein, LargeT, VP1, VP2, VP3
	Rearrangement	260	4703	699	38	1.6	All
	Rearrangement	312	4931	523	37	1.5	All
	Rearrangement	301	4778	665	32	1.3	All
	Rearrangement (inversion)	1857	5048	1,950	28	1.2	LargeT, SmallT, VP1
	Rearrangement	2862	5129	2,266	27	1.1	LargeT, SmallT
	Rearrangement	324	4443	1,023	26	1.1	Agnoprotein, LargeT, VP1, VP2, VP3
	Deletion	4851	5007	156	23	0.9	LargeT, SmallT
	Rearrangement	337	2519	2,181	23	0.9	Agnoprotein, VP1, VP2, VP3
	Deletion	1976	2481	505	22	0.9	VP1
	Rearrangement	1837	4991	1,988	20	0.8	LargeT, SmallT, VP1
	Deletion	3937	4869	932	17	0.7	LargeT, SmallT
	Rearrangement	232	1615	1,382	12	0.5	Agnoprotein, VP1, VP2, VP3
UBK282	Deletion	4421	4597	176	2,175	30.0	LargeT
	Deletion	1937	1941	4	1,290	17.8	VP1
	Rearrangement (inversion)	4247	5060	813	1,076	14.8	LargeT, SmallT
	Deletion	3781	3790	9	545	7.5	LargeT
	Deletion	3831	4018	187	503	6.9	LargeT
	Rearrangement (inversion)	4250	5057	807	475	6.6	LargeT, SmallT
	Rearrangement	4886	3850	1,037	422	5.8	All
	Rearrangement (inversion)	95	4789	440	381	5.3	All
	Deletion	2882	3538	656	294	4.1	LargeT
	Deletion	762	766	4	290	4.0	VP2
	Rearrangement	3663	5028	1,364	247	3.4	LargeT, SmallT
	Deletion	3540	3550	10	220	3.0	LargeT
	Deletion	1922	1929	7	191	2.6	VP1
	Rearrangement	4143	3506	638	188	2.6	All
	Rearrangement (inversion)	4246	5059	813	156	2.2	LargeT, SmallT
	Rearrangement (inversion)	4249	5056	807	135	1.9	LargeT, SmallT
	Rearrangement	4341	3001	1,341	134	1.9	All
	Deletion	4721	4894	173	130	1.8	SmallT
	Rearrangement (inversion)	2785	2859	74	79	1.1	LargeT
	Deletion	4626	4716	90	58	0.8	SmallT
	Rearrangement	3500	3494	7	52	0.7	All
	Rearrangement (inversion)	2764	5058	2,294	41	0.6	LargeT, SmallT
	Deletion	4364	4788	424	26	0.4	LargeT, SmallT
	Rearrangement (inversion)	4857	5109	252	23	0.3	LargeT, SmallT
	Deletion	4420	4610	190	20	0.3	LargeT
	Deletion	540	553	13	19	0.3	Agnoprotein
	Rearrangement	4665	3510	1,156	18	0.3	All
	Rearrangement	23	5122	36	15	0.2	All
UBK007—using Kapa HyperPrep Plus kit	Rearrangement	2805	5068	2,262	98	17.92	LargeT, SmallT
	Rearrangement	268	4628	763	90	16.45	All
	Deletion	236	346	110	43	7.86	None
	Deletion	275	428	153	20	3.66	Agnoprotein
UBK096—using Kapa HyperPrep Plus kit	Rearrangement (inversion)	192	4903	430	44	41.9	All
	Rearrangement (inversion)	4736	4905	169	35	33.33	LargeT, SmallT

The nucleotides present in the junctions in the BKPyV strains were separated by a range of 4 to 2,491 nucleotides (nt) (median, 628 nt). Notably, multiple unique junctions (median, 15; range, 2 to 40) were identified in each DVG-containing sample. All 12 BKPyV strains had junctions including the large T antigen, and in 10 of the strains, the most abundant deletion or rearrangement involved the large T antigen (Table 1). The most abundant junction in 11 of the BKPyV strains were internal deletions and, correspondingly, these DVGs were classified as deletion type. In the final strain, UBK096, the most abundant junction was an inversion rearrangement. In the 1 JCPyV strain with detectable junctions, 15 unique junctions, ranging from 285 to 2,302 nucleotides in length (median, 1,917 nt) were identified (Table 2). The most abundant junction was a rearrangement that spanned all three capsid proteins, VP1, VP2, and VP3. Other DVGs in the JCPyV present in specimen UBK292 were classified as deletion type.

**TABLE 2:** Rearragement and deletion junction positions and counts for JCPyV DVGs as detected by Geneious Prime using a threshold of at least 10 reads and frequency >10% compared to maximum depth

Sample	Junction type	Nucleotide start position	Nucleotide end position	Length (bp)	No. of junction reads	Reads with junction (%)	Genes affected
JCPyV in UBK292	Rearrangement	402	2576	2,173	508	12.48	Agnoprotein, VP1, VP2, VP3
	Rearrangement	425	2561	2,135	356	8.75	Agnoprotein, VP1, VP2, VP3
	Deletion	103	5121	285	351	8.63	All
	Rearrangement	386	4423	1,089	284	6.98	Agnoprotein, LargeT, VP1, VP2, VP3
	Rearrangement	2720	4739	2,018	162	3.98	LargeT, SmallT
	Rearrangement	2626	4582	1,955	46	1.13	LargeT, VP1
	Rearrangement	2640	4641	2,000	44	1.08	LargeT, SmallT, VP1
	Rearrangement	2794	4200	1,405	38	0.93	LargeT
	Rearrangement	430	4721	835	34	0.84	All
	Rearrangement	2186	4159	1,972	19	0.47	LargeT, VP1
	Rearrangement	334	4502	958	18	0.44	Agnoprotein, LargeT, VP1, VP2, VP3
	Rearrangement	2345	4648	2,302	17	0.42	LargeT, SmallT, VP1
	Deletion	4488	5034	546	14	0.34	LargeT, SmallT
	Rearrangement	287	4429	984	13	0.32	Agnoprotein, LargeT, VP1, VP2, VP3
	Rearrangement	1043	2961	1,917	11	0.27	LargeT, VP1, VP2, VP3

We confirmed that the observed junctions were not an artifact of our library preparation protocol by performing a second method for generating sequencing libraries on a subset of DVG-containing and DVG-negative samples. Identical junctions were found in the DVG-containing samples, and no new DVGs were found in DVG-negative samples using the different library preparation (Fig. S1). We further confirmed select junctions using specific PCR and Sanger sequencing (NCBI BioProject PRJNA657423).

### An elevated VP1-to-large T antigen ratio is observed in BKPyV strains containing defective viral genomes and having high viral loads.

We have previously shown that digital droplet PCR (ddPCR) is a cost-effective method to detect and confirm copy number alterations in cultured BKPyV and JCPyV (17). As many of the BKPyV DVGs had rearrangements and deletions spanning the large T antigen at a high frequency and comparatively fewer rearrangements and deletions spanning VP1 (Table 1), we speculated that DVG-containing samples should have a high number of VP1 copies relative to large T antigen copies. Thus, a VP1-to-large T antigen copy number ratio in DVG-containing samples should be greater than 1, reflecting the abundancy of large T antigen defective genomes, while samples without DVGs should have a ratio of 1, indicative of a lack of rearrangements and deletions in these genomes. To confirm our hypothesis, we performed ddPCR targeting the VP1 and large T antigen on the 12 BKPyV strains containing DVGs by deep sequencing and 21 BKPyV strains without DVGs by deep sequencing.

**FIG 3:**
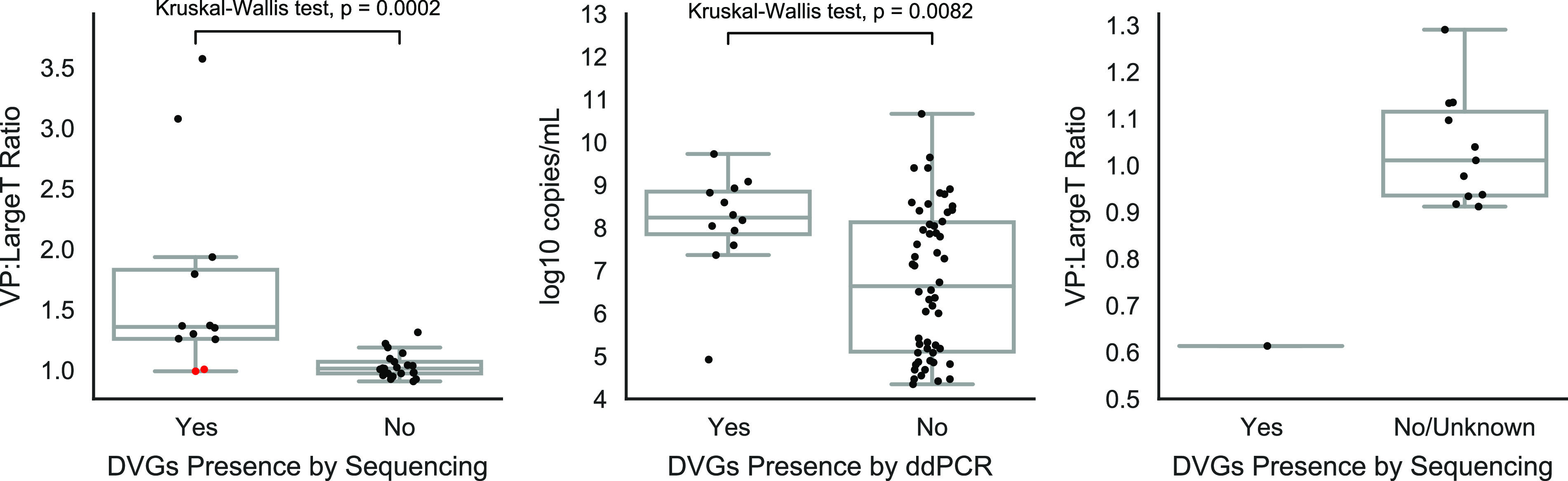
Confirmation of defective viral genomes using droplet digital PCR. (A) The copy number ratio for VP1 and large T antigen is plotted for 33 BKPyV isolates with and without DVGs determined by sequencing. Quartiles for each group are plotted in a box-and-whiskers plot, and error bars are 1.5-fold the interquartile range. Red dots indicate two strains (UBK265/UBK266) with DVGs that had a comparably low level of rearrangements (3 to 5%) at the target loci as determined by sequencing, consistent with their equivalent copy number measured by ddPCR. Statistical comparison was performed via Kruskal-Wallis test. (B) Log_10_ copies/ml viral load values in 66 BKPyV-positive specimens are displayed. The presence of DVGs was determined by ddPCR using a VP1/large T antigen ratio cutoff of 1.25. Statistical comparison was performed via Kruskal-Wallis test. (C) ddPCR VP1/large T antigen copy number ratios for 12 JCPyV-positive specimens are depicted. One JCPyV specimen contained a sequence-confirmed DVG, while 7 of the 11 other specimens lacked DVGs by sequencing, and the remaining had unknown DVG status.

Quantitative analysis with a ddPCR assay showed that the VP1-to-large T antigen copy number ratio was significantly higher in those strains with DVGs (mean, 1.69; range, 0.99 to 3.57) than in those with intact genomes (mean, 1.02; range, 0.91 to 1.31) (*P* = 0.0002, Kruskal-Wallis test; Fig. 3A). Of note, two strains with DVGs (UBK265/UBK266) had a comparably low level of rearrangements (3 to 5%) by sequencing at the exact loci interrogated by the ddPCR and had a ddPCR VP1/large T antigen ratio equivalent to 1. A VP1/large T antigen ratio cutoff of 1.25 separated the remaining 10 DVG-containing strains confirmed by sequencing from all strains without DVGs except UBK292.

We next used this ddPCR and established a ratio cutoff to screen 33 additional BKPyV-positive specimens for which high coverage genomes could not be recovered by shotgun sequencing due to low viral load. Notably, just one of the 33 low-viral-load samples had a VP1/large T antigen ratio greater than 1.25, consistent with low viral load infrequently containing DVGs. Furthermore, this analysis showed that specimens that tested positive for copy number alterations by ddPCR (VP1/large T antigen ratio of > 1.25) had a greater than 33-fold higher median viral load than those specimens that had a normal copy number ratio (median, 1.75 × 10^8^ copies/ml versus 5.30 × 10^6^ copies/ml; *P* = 0.0082, Kruskal-Wallis test) (Fig. 3B).

We next performed ddPCR targeting the VP1 and large T antigen on the JCPyV strain containing DVGs and 11 JCPyV-positive urine specimens, 7 of which did not contain DVGs by deep sequencing. The 11 JCPyV-positive strains had a mean VP1/large T antigen copy number ratio of 1.03 (range, 0.91 to 1.29), while the strain with the DVGs had a ratio of 0.61 (Fig. 3C), consistent with a copy number ratio of 0.58 by sequencing at the targeted loci.

### Phylogenetic analysis of BKPyV and JCPyV genomes reveals that strains containing defective viral genomes belong to multiple subgroups and are stable across time.

**FIG 4:**
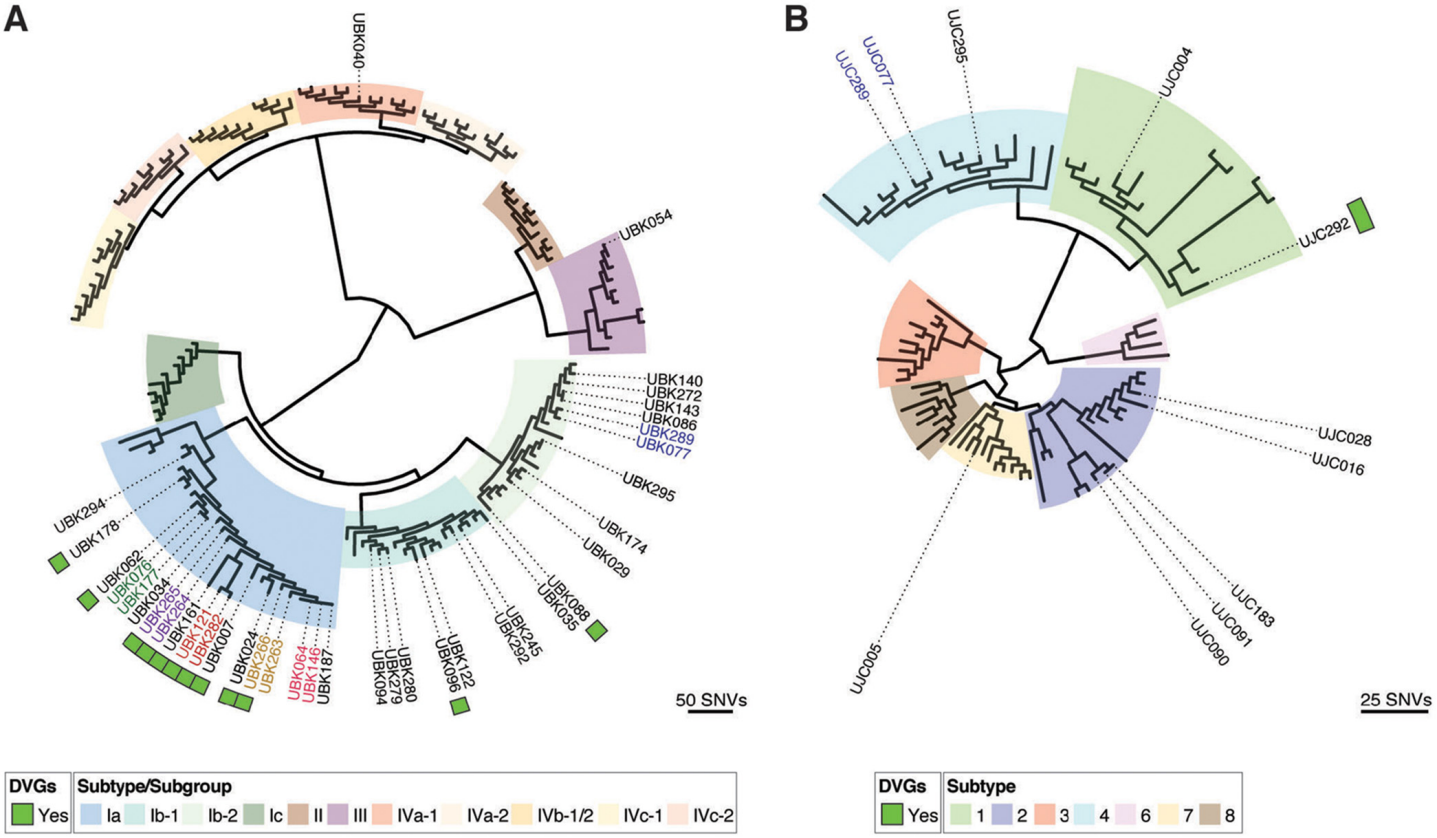
(A) Phylogenetic tree of 38 BKPyV consensus genomes recovered in this study along with 106 representative BKPyV genomes covering all 11 subtypes. BKPyV samples containing DVGs are indicated by a green square, and those samples longitudinally collected from the same individual are highlighted through coloring of the sample labels. (B) Phylogenetic tree of the 11 JCPyV genomes recovered in this study with 106 JCPyV covering 7 JCPyV subtypes. The samples containing DVGs are indicated with a green square.

We next assessed the genetic relatedness of the BKPyV strains that produced DVGs by performing a phylogenetic analysis with 106 representative BKPyV genomes and the 38 total BKPyV genomes recovered in this study (Fig. 4A). Of the 38 strains, 36 belonged to subtype I, 1 belonged to subtype III, and 1 belonged to subtype IV. These observations are consistent with subtype I being the most prevalent subtype in the United States and subtypes II to IV being less frequently detected in North America (33). Of the 36 subtype I strains, 18 belonged to subgroup Ia, 8 belonged to subgroup Ib-1, and 10 belonged to subgroup Ib-2. The 12 strains containing DVGs all belonged to subtype I. Ten of these strains belonged to subgroup Ia, 1 belonged to subgroup Ib-1, and 1 belonged to subgroup Ib-2.

Of the 38 BKPyV genomes recovered in this study, 6 pairs of isolates were collected from the same individuals (UBK064/UBK146, UBK077/UBK289, UBK076/UBK177, UBK121/UBK282, UBK264/UBK265, UBK263/UBK266), allowing us to look at longitudinal evolution of BKPyV DVGs. The first three of these pairs (UBK064/UBK146, UBK077/UBK289, UBK076/UBK177) did not contain any DVGs and were genetically identical across a longitudinal sampling time of 7, 487, and 21 days, respectively. Interestingly, in one pair collected 159 days apart, the first isolate, UBK263, only contained intact genomes, while the second isolate, UBK266, contained DVGs. Only 1 consensus single nucleotide variant (SNV) was recovered between these samples, which resulted in a coding change (Glu43Lys) in the agnoprotein. Notably, the BKPyV viral load increased by 57.5× between UBK263 and UBK266, suggesting that the increase in viral load may have contributed to the formation of DVGs. In the final two pairs (UBK121/UBK282 and UBK264/UBK265), both strains contained DVGs. UBK121 and UBK282 were collected 302 days apart and differed by 2 SNVs as well as a 39-bp insertion at the origin. One of the SNVs resulted in a coding change, Lys556Arg, in the large T antigen. UBK264 and UBK265 were collected 41 days apart and differed by 2 SNVs, both of which resulted in coding changes in the agnoprotein (Gln8Arg and Glu43Lys).

We then assessed the genetic relatedness of the 11 JCPyV strains sequenced in this study by performing a phylogenetic analysis with 64 representative JCPyV genomes (Fig. 4B). Two of the strains belonged to subtype 1, 5 belonged to subtype 2, 3 belonged to subtype 4, and 1 belonged to subtype 7. The one JCPyV strain containing DVGs belonged to subtype 1.

### BKPyV DVG-containing strains are more likely to contain unfixed mutations in the BC loop of VP1 than non-DVG containing strains.

The host DNA cytosine deaminase APOBEC3B has been shown to shape the intrahost evolution of BKPyV within the kidney (34). APOBEC3B targets cytosines contained within specific trinucleotide motifs (YCD) (35–37). In BKPyV, APOBEC3B recognition sites are present more often on the antisense DNA strand, and APOBEC3B-associated mutagenesis is speculated to contribute to the emergence of antibody escape mutations (36). The antisense DNA strand that encodes the BC loop, which is often a target of neutralizing antibodies, of the VP1 viral capsid protein, has a number of APOBEC3B recognition sites (36, 38, 39). We examined all 38 BKPyV strains for mutations that did not reach fixation (allelic frequency between 10 and 90%) within the BC loop of VP1 that could be attributed to APOBEC3B damage. Of the 38 BKPyV strains, 10 had an unfixed mutation potentially associated with APOBEC3B mutagenesis (Table 3). Four of these strains had a nephropathy-associated mutation, Glu73Gln, attributed to APOBEC3B activity. Interestingly, 8 of the 10 strains with APOBEC3B-associated mutations in the BC loop of VP1 contained DVGs. We then examined all 38 BKPyV strains for unfixed mutations in the BC loop that were unlikely to be associated with APOBEC3B mutagenesis. Three DVG-containing BKPyV strains had additional mutations in the BC loop of VP1, while two of the non-DVG containing strains had additional BC loop mutations. In total, 8 of the 12 (66.7%) DVG-containing BKPyV strains had unfixed BC loop mutations, while just 3 of the 26 (11.5%) DVG-containing strains had such mutations.

**TABLE 3:** Unfixed, nonsynonymous mutations located in the BC loop of VP1 identified in the BKPyV strains sequenced in this study^*a*^

Sample	Contains DVGs?	Amino acid mutation	Frequency	APOBEC3B-associated?	APOBEC3B trinucleotide mutation	Strand containing APOBEC3B recognition site
UBK024	Yes	Glu60Gln	15.9	Yes	TCT → TGT	Antisense
		66insSerLeu	31.4	No	NA	NA
		Lys71Asn	15.3	No	NA	NA
		Asn79Ser	17.1	No	NA	NA
		Ser80Thr	15.6	No	NA	NA
UBK029	No	Lys74Asn	35.3	No	NA	NA
UBK062	Yes	Leu68Val	89.7	Yes	TCT → TGT	Sense
		Glu82Gln	40.5	Yes	TCT → TGT	Antisense
UBK096	Yes	Glu73Gln	38.2	Yes	TCA → TGA	Antisense
		Glu73Lys	25.9	Yes	TCA → TTA	Antisense
		Glu82Gln	11.9	Yes	TCT → TGT	Antisense
UBK121	Yes	Asp58Asn	12.9	No	NA	NA
		Glu60Gln	36.2	Yes	TCT → TGT	Antisense
		Lys69Gln	36.8	No	NA	NA
		Val72Ile	35.5	No	NA	NA
		Glu82Gln	85	Yes	TCT → TGT	Antisense
		Gln82His	14.9	Yes	TCT → TGT	Antisense
UBK122	No	Ala72Val	17.7	No	NA	NA
		Glu73Gln	46.3	Yes	TCA → TGA	Antisense
UBK161	Yes	Gln82His	22.5	Yes	TCT → TGT	Antisense
UBK177	No	Glu73Gln	15.1	Yes	TCA → TGA	Antisense
UBK178	Yes	Glu73Gln	13.6	Yes	TCA → TGA	Antisense
		Glu82Gln	88.6	Yes	TCT → TGT	Antisense
UBK266	Yes	Asp60Asn	31.6	Yes	TCT → TTT	Antisense
UBK282	Yes	Asp58Asn	18.9	No	NA	NA
		Glu60Gln	44.1	Yes	TCT → TGT	Antisense
		Lys69Gln	45.2	No	NA	NA
		Val72Ile	43.1	No	NA	NA
		Ser78Asn	10	No	NA	NA
		Gln82His	32.9	Yes	TCT → TGT	Antisense

a NA, not applicable.

### Persons with DVG-containing viruses are more likely to have changes to immunosuppressive regimens.

Clinical information was available for 31 individuals with BK viruria, including 22 individuals without any samples containing DVGs and 9 individuals with DVGs identified in at least one clinical sample (Tables 4 and 5). Both the DVG-negative and DVG-containing groups were similar in age (mean, 55 ± 16.3 versus 56 ± 17.7 years) and proportion of females (54.5% and 55.6%), respectively. The clinical setting under which all individuals developed BK viruria was related to immunosuppression secondary to organ transplant. Renal transplant was the most common indication for both groups, with peripheral blood stem cell and multiorgan (kidney and pancreas or kidney and liver) transplants representing a smaller percentage. Comparison of immunosuppressive regimens between DVG groups demonstrated that DVG-containing individuals more often had their dose of mycophenolate stopped or changed to leflunomide. Changes in therapy for the DVG-containing group are perhaps related to their higher viral loads.

**TABLE 4:** Clinical characteristics of the individuals in this study

Characteristic	DVG (–)	DVG (+)
No. of patients	22	9
Age (yrs), mean (SD)	55 (16.3)	56 (17.7)
Sex		
Female (*n*)	12	5
Male (*n*)	10	4
Diagnosis		
End-stage renal disease (*n*)	19	8
Hematological malignancy (*n*)	3	1
Organ transplant		
Kidney (*n*)	15	8
Peripheral blood stem cell (*n*)	3	1
Kidney and pancreas (*n*)	3	0
Kidney and liver (*n*)	1	0
Change in management		
No change (*n*)	10	2
Reduce mycophenolate (*n*)	4	0
Stop mycophenolate (*n*)	6	3
Change to leflunomide (*n*)	2	4
Received intravenous immunoglobulin (*n*)	3	2

**TABLE 5:** Laboratory and pathology information by DVG status

Biopsy	DVG (–)			DVG (+)		
	Pos	Neg	NR^*a*^	Pos	Neg	NR^*a*^
Evidence of virus (SV40 IHC), n	2	3	17	1	4	4
Evidence of rejection, n	2	6	14	1	6	2
Serology						
Recipient CMV, n	13	8	1	7	2	0
Donor CMV, n	11	9	2	1	8	0
Recipient EBV, n	17	3	2	8	0	1

a Pos, positive; neg, negative; NR, not reported;

DVG-containing individuals were more likely to undergo renal biopsy (7 of 9 versus 8 of 22). However, histologic evidence of kidney injury or immunohistochemical staining for the large T antigen was rarely seen (1 and 4 individuals, respectively). Pretransplant serologies demonstrated the majority of individuals in both groups to be cytomegalovirus (CMV) and Epstein-Barr virus (EBV) positive. In the DVG-containing group, individuals more often received organs from donors who were CMV-negative.

## DISCUSSION

Here, we report the presence of polyomavirus defective genome segments in clinical specimens and observe the diversity of DVG populations naturally generated across individuals and within a host over the course of infection. Specifically, by deep sequencing, we identified multiple BKPyV strains and one JCPyV strain that contained large deletions or rearrangement junctions in their genomes. Although these junctions have been observed in all six gene segments, most were present in the large T antigen, which is consistent with reports from other viruses in which DVGs most commonly occur in replication-related genes (13). These observations also are consistent with the characteristic features of the polyomavirus DVGs found in culture—major internal deletions with retention of regions that are essential for genome packaging (4, 13, 24).

DVG formation is thought to be a form of viral parasitism, which occurs when multiple viruses infect the same cell (4, 5, 13, 24). Our finding that DVGs were more likely to be present when polyomavirus was present at high copy numbers is consistent with this model. A dramatic increase in viral load was temporally also associated with the only longitudinal specimens in which DVGs newly arose. Alternatively, DVGs may enhance the pathogenicity of the virus by assisting the virus to evade the host’s immune response, as is observed with the *Turnip crinkle virus* (2, 3). Consistent with this hypothesis, we observed a higher frequency of unfixed mutations in the BC loop of the capsid protein VP1 in DVG-containing BKPyV strains than in those strains without DVGs. Interestingly, many of these BC loop mutations could have arisen through APOBEC3B-associated mutagenesis, which has been speculated to produce antibody escape mutations in BKPyV (36, 38, 39). These unfixed mutations in DVGs may provide the virus with unique and mosaic capsids that are not targeted by the host’s existing set of polyomavirus-directed neutralizing antibodies. In addition, the higher frequency of potential APOBEC3B-associated mutations in DVG-containing strains may suggest that APOBEC3B activity could be driving the formation of DVGs. Alternatively, the bidirectional replication mechanism of polyomaviruses combined with decatenation of linked circular genomes would provide several opportunities for recombination and generation of deletions and other large-scale rearrangements (14, 40, 41).

We also examined the evolution of BKPyV in a small subset of individuals that had longitudinal specimens available. The recovery of 1 to 2 SNVs in specimens taken from persons with longitudinal samples spaced between 1 month and 1 year apart is consistent with the relatively high intrahost evolutionary rate of polyomaviruses, previously measured at 4.9 × 10^−4^ to 1.2 × 10^−3^ substitutions per site per year (42). Given that we only detected one newly generated DVG in this study, more work is required to uncover the determinants and rate of DVG generation in polyomaviruses.

One main limitation of our sequencing approach was the use of short-read sequencing, which may have affected our measurements of DVGs in each specimen and did not allow us to link rearrangements across the multiple populations of virus present within the viral population. Recent work in polyomaviruses has demonstrated the promise of long-read sequencing to resolve complex populations of polyomaviruses (43, 44). However, the limited total DNA contained in these samples complicates the use of long-read approaches given the large-scale rearrangements recovered and requirement for multiple cycles of PCR. We also quantitated rearrangements as a percentage of maximum coverage, which may bias quantitation depending on local variations in copy number. We bulwarked our sequencing approach by using multiple library preparations of the same sample to illustrate that deletions and rearrangements recovered were not due to library generation.

Of note, it has yet to be established whether these DVGs identified from the clinical specimens interfere with the replication of the full-genome virus *in vitro*, and the role of DVGs in natural polyomavirus infections has yet to be addressed. The deletions and rearrangements recovered in our study were generally quite large and affected proteins and domains required for viral replication, consistent with their defective nomenclature. Further studies focusing on the function of specific rearrangements found in polyomavirus DVGs through reverse genetics and cell culture-based approaches are required to help understand their role in replication interference.

In conclusion, by demonstrating the existence of polyomavirus DVGs in human specimens, we extend this broad property of viral evolution to DNA viruses as they exist in human hosts. The polyomaviruses containing DVGs recovered in this study generally had significantly higher copy number of capsid genes to replication genes. Further work is required to determine whether these genetic copy alterations result in differences in protein levels in human samples that could potentially affect pharmacodynamic properties and efficacy profiles of capsid-directed therapies for polyomaviruses.

## MATERIALS AND METHODS

### Clinical testing and next-generation sequencing.

This work was approved by the University of Washington Institutional Review Board (STUDY00000408). Urine samples were collected from individuals suspected to have a BKPyV or JCPyV infection, and clinical testing was performed at the University of Washington Virology Laboratory using the previously described quantitative PCR assays (16, 45).

BKPyV- or JCPyV-positive urine samples were first filtered using a 0.22-μM filter, and DNA was extracted from these samples using the Quick-DNA viral kit (Zymo). Sequencing libraries were constructed from 2 μl of DNA using the Nextera XT kit (Illumina) and cleaned with 0.6× volumes of AMPure XP beads (Beckman Coulter). For four samples, a second sequencing library was created using the KAPA HyperPrep Plus kit (Roche) and purified with 0.8× volumes of AMPure XP beads. The resulting libraries were then sequenced on 1 × 192-bp or 2 × 300-bp Illumina MiSeq runs.

### Identification of defective viral genomes.

Sequencing reads were adapter- and quality-trimmed using Trimmomatic v0.38 (46) and mapped to the BKPyV (NC_001538.1) or JCPyV (NC_001699.1) reference genome. Consensus genomes were manually called, and sequencing reads were then mapped to the respective consensus genome in Geneious Prime (47), with the structural variant, insertion, and deletion detection setting enabled. Because of the high proportion of small deletions contained around the origin and upstream of the agnoprotein, those junctions entirely contained between nucleotides 0 to 300 were discarded and not included in downstream further analysis. Each of the junctions reported here was supported by a minimum of 10 sequencing reads. In addition, we confirmed the presence of the highest-abundance junction identified in each sample using DI-tector (48).

Next, we confirmed the presence of these junctions in a subset of these samples through PCR and Sanger sequencing. PCR amplification was performed using the CloneAmp HiFi Premix (TaKaRa) and the primers listed in Table 6 under the following conditions: hold at 98°C for 2 min, followed by 35 cycles of 98°C for 10 s, 61°C for 15 s and 72°C for 30 s, followed by a final extension at 72°C for 5 min. PCR products were run on a 1.5% agarose gel and extracted using the QIAquick gel extraction kit (Qiagen). Sanger sequencing was performed with the primers listed in Table 6 by Genewiz, Inc.

**TABLE 6:** Primer and probe sequences used in this study

Purpose	Name	Sequence (5′ → 3′)
For Sanger sequencing	BK-DIP-F2	GATGGGCAGCCTATGTATGG
	BK-DIP-R2	GCAATGGTGGGTCCAAATAATTG
	BK62-DIP-1	GGCTGAAGTATCTGAGACTTGG
	BK62-DIP-2	CTTGCCTGCTTTGCTGTGTAT
For ddPCR	BKV_VP_Fwd	GCCCCAGGAGGTGCTAATC
	BKV_VP_Rev	CAGGCCTAGAAGTAAAGGCAACA
	BKV_VP_Probe	**Fam**_AGAACTGCTCCTCAATG-**MGB**
	BKV_largeT_Fwd	TTATCTCAGAATCCAGCCTTTCCT
	BKV_largeT_Rev	GGCCTGTAGCTGATTTTGCAA
	BKV_largeT_Probe	**Vic**_CCATTCAACAATTCTAG-**MGB**
	JCV_VP_Fwd	CACAGAGCACAAGGCGTACC
	JCV_VP_Rev	AAGCAACACTGTTGTGGCAG
	JCV_VP_Probe	**Fam**_TTCCTGATCCCACC_**FQ**
	JCV_LargeT_Fwd	CCAGTGCCTTTTACATCCTC
	JCV_LargeT_Rev	GGCCAATAGACAGTGGCAA
	JCV_LargeT_Probe	**Hex**_ATCAAGTAAAGCTGCAGCT_**FQ**

### Droplet digital PCR detection of defective viral genomes.

ddPCR was performed using the Bio-Rad QX100 system (Bio-Rad, Hercules, CA, USA) and QuantaSoft for data analysis. There are two sets of primers and probes targeting the VP1 and large T antigen regions, respectively (Table 6). Each reaction was performed with Bio-Rad ddPCR supermix for probes with the final concentration of primers at 900 nM and probes at 250 nM and 25 units of HindIII (New England Biolabs). Plasmid BK Dunlop and JC Mad-1 were gifts from Peter Howley (Addgene plasmids no. 25466 and no. 25626) and were used as positive controls for 1:1 VP1/large T-antigen copy number. After droplet generation, droplets were transferred to a 96-well PCR plate and amplified on a 2720 thermal cycler (Applied Biosystems) with the following thermocycling parameters: 94°C for 10 min, followed by 40 cycles of 94°C for 30 s and 60°C for 1 min, and 98°C hold for 10 min. After the thermal cycling, the plate was transferred to a droplet reader. The QuantaSoft software was used for data analysis.

### Phylogenetic, metagenomic, and unfixed variant analysis.

We downloaded all 510 complete BKPyV genomes from NCBI GenBank (accessed 16 August 2020) and removed any duplicate genomes from these data set to obtain 402 unique BKPyV genomes. These 402 BKPyV genomes were then classified into the 11 VP1 sequence subtypes and subgroups with BKTyper 1.0 (49). We next randomly selected 10 genomes from each of these subtypes or subgroups for inclusion in our phylogenetic analysis. The 106 representative BKPyV genomes and the 38 complete BKPyV genomes obtained in this study were aligned with MAFFT v7.429 (50). A phylogenetic tree was generated from this alignment using RaxML v8.2.11 (51) and visualized with ggtree (52).

To perform the JCPyV phylogenetic tree, we downloaded all 696 complete JCPyV genomes from NCBI GenBank (accessed 16 January 2021) and removed any duplicate genomes to obtain 565 unique JCPyV genomes. These genomes were then classified into the previously defined JCPyV subtypes (53) based on their VP1 sequence using a custom Python script. We then randomly selected genomes from each subtype. An alignment and a phylogenetic tree were generated as described above with these 64 representative JCPyV genomes and the 11 JCPyV genomes recovered in this study.

Metagenomic analysis was performed as previously described (54), using the metagenomic classifier CLOMP (https://github.com/rcs333/CLOMP). Counts were normalized between samples, and classifications were expressed as reads per million (RPM). Taxonomic classifications of each read were assigned to the most specific NCBI taxonomy ID possible. As such, reads aligning to two or more reference genomes within a taxonomical classification were assigned to the next lowest taxonomical category. Any reads assigned to environmental or artificial sequences were discarded. Reads that matched equally well to two or more different domains were categorized as “unclassified.”

Unfixed variants in the BC loop of the VP1 (amino acids 57 to 89 in the BKPyV reference genome [NC_001538.1]) were detected by mapping sequencing reads to the consensus BKPyV genome obtained for each sample in Geneious Prime (38, 47). A minimum sequencing depth of 10× and allele frequency of greater than 10% were used for calling variants. We considered unfixed variants to have an allele frequency less than 90%.

### Data availability.

Sequencing reads and consensus genomes are available under NCBI BioProject PRJNA657423; GenBank accession numbers MW587957 to MW587964, MW587966 to MW587994, and MW588006; and Sequence Read Archive accession numbers SRR13239807 to SRR13239811, SRR13239816 to SRR13239829, SRR13239831 to SRR13239851, SRR13239853 to SRR13239855, SRR13680575, and SRR13680576 (see Table 7).

**TABLE 7:** Accession numbers for samples sequenced in this study^*a*^

Sample ID	BKPyV Genome ID	BKPyV GenBank accession no.	JCPyV genome ID	JCPyV GenBank accession no.	Library prepn method 1	SRA accession no. 1	Library prepn method 2	SRA accession no. 2
UBK007	UBK007	MW587957	NA	NA	Nextera XT	SRR13239855	KAPA HyperPrep Plus	SRR13239815
UBK016	NA	NA	UJC016	MW587997	Nextera XT	SRR13239854
UBK024	UBK024	MW587958	NA	NA	Nextera XT	SRR13239843
UBK028	NA	NA	UJC028	MW587998	Nextera XT	SRR13239832
UBK029	UBK029	MW587959	NA	NA	Nextera XT	SRR13239821
UBK034	UBK034	MW587960	NA	NA	Nextera XT	SRR13239811
UBK035	UBK035	MW588006	NA	NA	Nextera XT	SRR13239810
UBK040	UBK040	MW587961	NA	NA	Nextera XT	SRR13239809
UBK054	UBK054	MW587962	NA	NA	Nextera XT	SRR13239808
UBK062	UBK062	MW587963	NA	NA	Nextera XT	SRR13239807
UBK064	UBK064	MW587964	NA	NA	Nextera XT	SRR13239853
UBK076	UBK076	MW587966	NA	NA	Nextera XT	SRR13239851
UBK077	UBK077	MW587967	UJC077	MW587999	Nextera XT	SRR13239850
UBK086	UBK086	MW587968	NA	NA	Nextera XT	SRR13239849	KAPA HyperPrep Plus	SRR13239814
UBK088	UBK088	MW587969	NA	NA	Nextera XT	SRR13239848
UBK090	NA	NA	UJC090	MW588000	Nextera XT	SRR13239847
UBK091	NA	NA	UJC091	MW588001	Nextera XT	SRR13239846
UBK094	UBK094	MW587970	NA	NA	Nextera XT	SRR13239845	KAPA HyperPrep Plus	SRR13239813
UBK096	UBK096	MW587971	NA	NA	Nextera XT	SRR13239844	KAPA HyperPrep Plus	SRR13239812
UBK121	UBK121	MW587972	NA	NA	Nextera XT	SRR13239842
UBK122	UBK122	MW587973	NA	NA	Nextera XT	SRR13239841
UBK140	UBK140	MW587974	NA	NA	Nextera XT	SRR13239840
UBK143	UBK143	MW587975	NA	NA	Nextera XT	SRR13239839
UBK146	UBK146	MW587976	NA	NA	Nextera XT	SRR13239838
UBK161	UBK161	MW587977	NA	NA	Nextera XT	SRR13239837
UBK174	UBK174	MW587978	NA	NA	Nextera XT	SRR13239836
UBK177	UBK177	MW587979	NA	NA	Nextera XT	SRR13239835
UBK178	UBK178	MW587980	NA	NA	Nextera XT	SRR13239834
UBK183	NA	NA	UJC183	MW588002	Nextera XT	SRR13239833
UBK187	UBK187	MW587981	NA	NA	Nextera XT	SRR13239831
UBK245	UBK245	MW587982	NA	NA	Nextera XT	SRR13239829
UBK263	UBK263	MW587983	NA	NA	Nextera XT	SRR13239828
UBK264	UBK264	MW587984	NA	NA	Nextera XT	SRR13239827
UBK265	UBK265	MW587985	NA	NA	Nextera XT	SRR13239826
UBK266	UBK266	MW587986	NA	NA	Nextera XT	SRR13239825
UBK272	UBK272	MW587987	NA	NA	Nextera XT	SRR13239824
UBK279	UBK279	MW587988	NA	NA	Nextera XT	SRR13239823
UBK280	UBK280	MW587989	NA	NA	Nextera XT	SRR13239822
UBK282	UBK282	MW587990	NA	NA	Nextera XT	SRR13239820
UBK289	UBK289	MW587991	UJC289	MW588003	Nextera XT	SRR13239819
UBK292	UBK292	MW587992	UJC292	MW588004	Nextera XT	SRR13239818
UBK294	UBK294	MW587993	NA	NA	Nextera XT	SRR13239817
UBK295	UBK295	MW587994	UJC295	MW588005	Nextera XT	SRR13239816
UJC004	NA	NA	UJC004	MW587995	Nextera XT	SRR13680576
UJC005	NA	NA	UJC005	MW587996	Nextera XT	SRR13680575

a NA, not applicable.
