# Supplementary material for: In Vivo Generation of BK and JC Polyomavirus Defective Viral Genomes in Human Urine Samples Associated with Higher Viral Loads
Source: J Virol. 2021 May 24;95(12):e00250-21. doi: 10.1128/JVI.00250-21 (PMC8316075; doi:10.1128/JVI.00250-21)
Supplement: Supplementary file 1 [file jvi.00250-21-s0001.pdf]

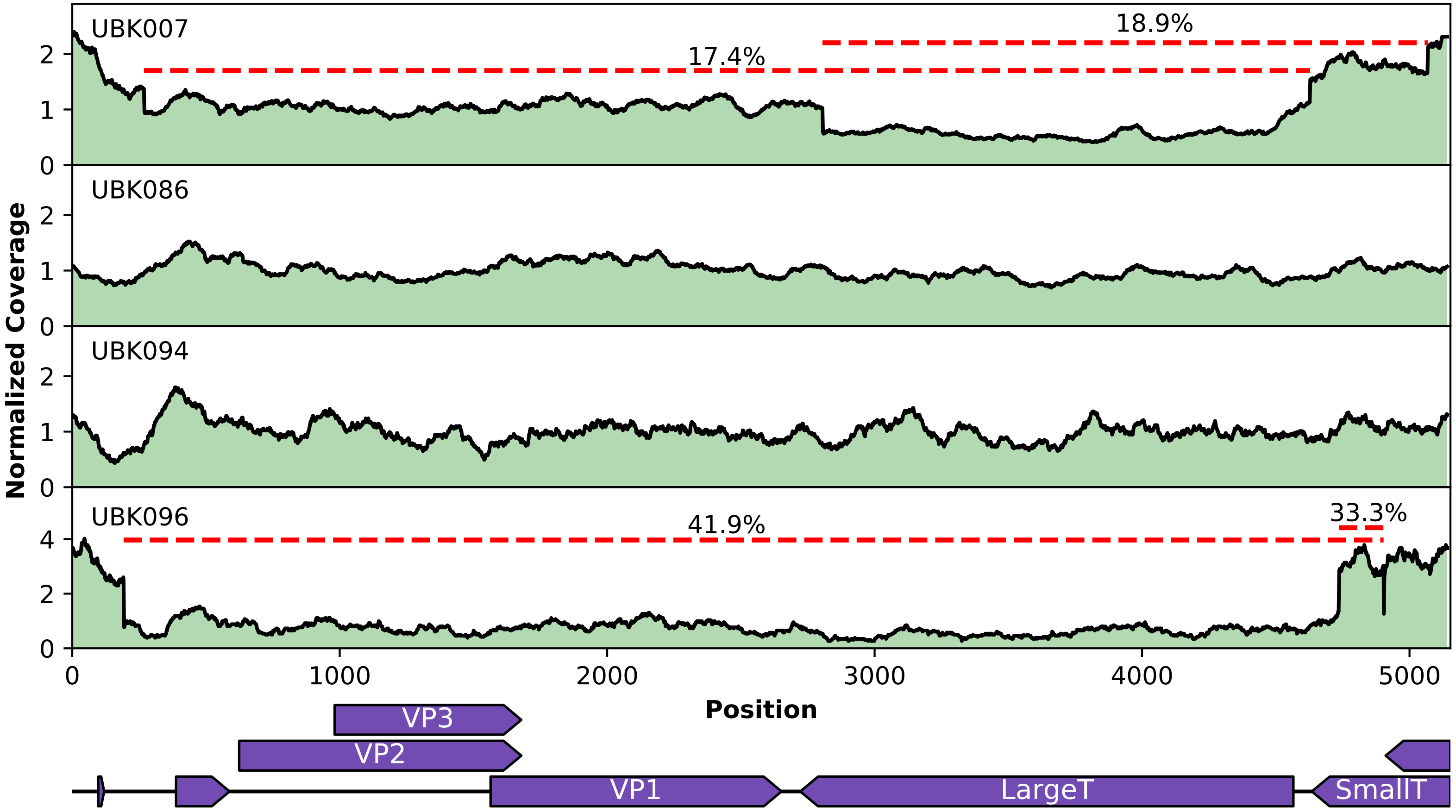

Figure S1. Coverage maps of two DVG-containing BKPyV strains, UBK007 and UBK096, and two DVG-negative BKPyV strains, UBK086 and UBK094, using KAPA HyperPlus library preparation kit as an alternative to the NexteraXT library preparation demonstrate reproducibility of the DVGs are not an artefact of a particular library preparation method. Coverage maps were generated in the same fashion as Figure 2.

Table S1A/B/C. Sample, sequencing, and ddPCR metadata for BKPyV or JCPyV positive samples.

Sample	BK virus Copies/mL	Total Reads	Reads on BK	% Reads on BK	Max Coverage	Mean Coverage	Reads containing DVGs	Cumulative DVG Depth as % of Max Depth
UBK007	8.40E+08	149501	77257	51.68	3692	1857	2425	65.68
UBK024	6.60E+08	192119	70517	36.7	2187	1521	1887	86.28
UBK029	2.60E+07	649561	142394	21.92	12263	5134	193	1.57
UBK034	3.60E+08	39253	30694	78.2	1250	953	30	2.4
UBK035	3.20E+08	160315	64747	40.39	5025	2225	466	9.27
UBK040	6.60E+07	454964	537	0.12	46	16	0	0
UBK054	2.50E+08	3734	1866	49.97	138	85	0	0
UBK062	1.50E+08	960280	264702	27.57	14747	6844	11948	81.02
UBK064	1.20E+08	55991	5872	10.49	602	193	0	0
UBK076	2.30E+08	259367	7021	2.71	577	209	0	0
UBK077	1.10E+06	356077	4558	1.28	917	166	24	2.62
UBK086	4.20E+09	206655	109447	52.96	4112	2703	0	0
UBK088	1.10E+08	161221	4806	2.98	602	157	553	91.86
UBK094	6.50E+08	189166	15495	8.19	632	491	0	0
UBK096	1.20E+09	96163	9507	9.89	972	258	926	95.27
UBK121	2.00E+08	227510	126173	55.46	10835	4226	25328	233.76
UBK122	3.90E+08	110338	52240	47.35	1648	1255	115	6.98
UBK140	1.40E+07	82523	63431	76.86	4297	2357	91	2.12
UBK143	2.30E+08	146479	81843	55.87	4760	2997	0	0
UBK146	7.20E+07	235888	2899	1.23	319	95	0	0
UBK161	3.90E+07	113331	81336	71.77	5342	2975	1982	37.1
UBK174	2.10E+07	214644	75314	35.09	6220	2803	29	0.47
UBK177	2.60E+08	172963	102536	59.28	8457	3404	109	1.29
UBK178	2.30E+07	183899	131535	71.53	10060	4987	3556	35.35
UBK187	6.20E+07	219958	4068	1.85	272	104	0	0
UBK245	1.10E+08	310124	19406	6.26	1331	656	0	0
UBK263	8.00E+08	27104	22201	81.91	1300	768	0	0
UBK264	3.90E+08	306055	2786	0.91	199	79	26	13.07
UBK265	6.10E+08	364638	165870	45.49	10124	5307	2111	20.85
UBK266	4.60E+10	36444	28420	77.98	2442	1346	547	22.4
UBK272	1.40E+08	342581	20328	5.93	1416	585	0	0
UBK279	8.90E+07	214168	134377	62.74	6054	4106	23	0.38
UBK280	8.90E+07	9608	5333	55.51	1053	180	0	0
UBK282	8.60E+07	106983	70023	65.45	7257	2127	9203	126.82
UBK289	5.80E+08	129976	1798	1.38	218	55	0	0
UBK292	5.30E+09	268089	70544	26.31	7643	2525	192	2.51
UBK294	2.50E+09	200066	124375	62.17	10216	3781	526	5.15
UBK295	4.40E+09	135271	118151	87.34	13363	3625	74	0.55

Sample	BK virus Copies/mL	Total Reads	Reads on JC	% Reads on JC	JCPyV Max Coverage	JCPyV Mean Coverage	Reads containing JCPyV DVGs	% Reads with JC DVGs over Max Coverage
UBK016	6.30E+04	1085885	79159	7.29	5159	2946	95	1.84
UBK028	1.50E+05	519556	13668	2.63	567	237.5	0	0
UBK077	1.10E+06	356077	16444	4.62	1271	614.1	76	5.98
UBK090	6.80E+05	370671	10237	2.76	644	323.1	0	0
UBK091	7.30E+04	1380762	1118	0.08	64	29.7	0	0
UBK183	2.60E+05	85548	2237	2.61	197	83.1	0	0
UBK289	5.80E+08	129976	635	0.49	84	19.5	0	0
UBK292	5.30E+09	268089	26843	10.01	4069	904	1915	47.06
UBK295	4.40E+09	135271	31392	23.21	8077	1032.6	0	0
UJC004	-	10294197	933077	9.06	60870	23951	0	0
UJC005	-	3764470	2254	0.06	230	60	0	0

Sample	BK virus Copies/mL	VP1/Large T antigen ddPCR ratio	DVGs by Sequencing	DVGs by ddPCR
UBK007	8.40E+08	1.79	Yes	Yes
UBK010	1.90E+07	0.93		No
UBK012	2.60E+04	1.10		No
UBK016	6.30E+04	1.04		No
UBK018	3.50E+06	1.03		No
UBK024	6.60E+08	1.35	Yes	Yes
UBK028	1.50E+05	1.17		No
UBK029	2.60E+07	1.04	No	No
UBK032	1.90E+05	1.08		No
UBK034	3.60E+08	0.96	No	No
UBK035	3.20E+08	1.22	No	No
UBK038	2.10E+06	1.00		No
UBK048	2.90E+04	1.06		No
UBK054	2.50E+08	1.14	No	No
UBK060	7.50E+07	1.04		No
UBK062	1.50E+08	3.08	Yes	Yes
UBK064	1.20E+08	0.97	No	No
UBK077	1.10E+06	0.97	No	No
UBK082	2.30E+06	1.06		No
UBK088	1.10E+08	3.57	Yes	Yes
UBK091	7.30E+04	1.05		No
UBK094	6.50E+08	1.09	No	No
UBK096	1.20E+09	1.37	Yes	Yes
UBK098	7.80E+04	1.08		No
UBK105	2.90E+04	1.00		No
UBK106	3.20E+06	0.95		No
UBK109	1.50E+05	1.00		No
UBK116	2.20E+04	0.92		No
UBK118	1.30E+07	1.00		No
UBK121	2.00E+08	1.37	Yes	Yes
UBK122	3.90E+08	1.04	No	No
UBK123	4.10E+07	1.01		No
UBK124	1.80E+05	0.95		No
UBK132	2.10E+05	1.06		No
UBK134	4.80E+04	1.05		No
UBK138	7.10E+04	0.96		No
UBK139	5.30E+06	1.05		No
UBK140	1.40E+07	0.98	No	No
UBK142	1.50E+06	0.99		No

UBK143	2.30E+08	0.98	No	No
UBK146	7.20E+07	1.07	No	No
UBK152	1.20E+05	1.09		No
UBK155	3.50E+04	0.89		No
UBK161	3.90E+07	1.26	Yes	Yes
UBK163	8.30E+04	1.80		Yes
UBK174	2.10E+07	0.91	No	No
UBK177	2.60E+08	0.95	No	No
UBK178	2.30E+07	1.30	Yes	Yes
UBK183	2.60E+05	1.12		No
UBK187	6.20E+07	1.02	No	No
UBK192	4.80E+04	0.93		No
UBK199	6.50E+04	1.00		No
UBK207	1.20E+05	1.07		No
UBK245	1.10E+08	0.93	No	No
UBK262	1.00E+06	0.99		No
UBK263	8.00E+08	1.01	No	No
UBK264	3.90E+08	1.25	Yes	Yes
UBK265	6.10E+08	1.01	Yes	No
UBK266	4.60E+10	0.99	Yes	No
UBK272	1.40E+08	1.02	No	No
UBK279	8.90E+07	1.19	No	No
UBK282	8.60E+07	1.93	Yes	Yes
UBK290	2.50E+09	0.82		No
UBK292	5.30E+09	1.31	No	Yes
UBK294	2.50E+09	1.01	No	No
UBK295	4.40E+09	0.93	No	No

Table S2. RPM values of uropathogenic bacteria and fungi in 46 BKPyV- or JCPyV-positive samples. Uropathogenic bacteria were considered present when any bacterial species had greater than 10 RPM.

Sample	*S.aureus*	*S. saprophyticus*	*Enterobacter*	*Citrobacter*	*Klebsiella*	*E.coli*	*P. aeruginosa*	*E. faecalis*	*E. faecium*	*P. stuartii*	*P. mirabilis*	*Candida*	Bacteria_Sum
UBK007	11	0	0	0	0	0	0	0	0	0	0	0	11
UBK016	0	0	77	68	14	5	24	0	0	0	0	0	188
UBK024	0	0	0	0	0	0	0	0	0	0	0	11	11
UBK028	0	0	0	0	0	0	2	4	0	0	0	15	22
UBK029	0	0	0	0	69	5	3	2	0	0	0	0	79
UBK034	29	29	58	0	29	0	0	0	0	0	0	29	173
UBK035	0	0	145	0	100	22	22	0	0	0	0	11	301
UBK040	18	0	3	0	13	0	11	0	0	3	0	45	92
UBK054	0	0	0	0	2904	953	67	15192	119	0	0	0	19236
UBK062	0	0	0	0	148	295	295	0	0	0	0	148	885
UBK064	0	0	2	0	5	0	0	0	0	0	0	0	7
UBK076	0	10	10	0	36	0	41	7777	10	0	0	0	7886
UBK077	0	0	0	8	8	348	8	0	0	0	0	4	376
UBK086	0	0	0	0	0	8	0	0	0	0	0	0	8
UBK088	0	0	0	0	23	262	31	0	0	0	0	0	316
UBK090	5	0	0	5	5	5	0	0	0	0	0	10	31
UBK091	1	0	0	0	0	0	1	0	0	0	0	2	4
UBK094	0	6	6	30	12	0	12	0	0	0	0	6	73
UBK096	46	0	9	0	9	28	46	0	0	0	0	55	194
UBK121	0	0	0	0	185	5	0	11	0	0	0	0	201
UBK122	45	11	0	22	206278	112	0	22	11	0	0	0	206502
UBK140	0	0	0	0	0	349	0	0	0	0	0	0	349
UBK143	0	0	0	7	0	0	0	0	0	0	0	0	7
UBK146	0	0	0	0	0	5	0	0	0	0	0	0	5
UBK161	0	0	0	0	0	0	88	0	0	0	0	0	88
UBK174	0	0	0	0	0	0	1058	5	0	0	0	0	1063
UBK177	0	0	0	0	12	0	6	824	0	0	0	0	843
UBK178	0	6	0	30	0	304	480	0	0	0	0	0	821
UBK183	0	0	14	376	1431	463	0	0	0	0	0	0	2284
UBK187	6	0	6	0	0	0	0	12	0	0	0	0	24
UBK245	11	0	0	7	0	0	0	18	249	0	0	0	286
UBK263	0	0	42	0	0	0	84	0	0	0	0	84	211
UBK264	0	0	0	0	2	5	1	0	1	0	1	0	11
UBK265	0	0	0	0	0	3	0	0	0	0	0	0	3
UBK266	0	0	0	0	193	0	29	0	0	0	0	0	222
UBK272	0	0	0	22	14	22	7	51	6220	0	521	0	6857
UBK279	19	6	25	13	57	13	76	25	6	13	0	13	268
UBK280	0	0	0	0	0	0	0	0	0	0	0	0	0
UBK282	0	0	0	0	36	0	0	0	0	0	0	0	36
UBK289	0	0	0	0	0	0	0	0	0	0	0	0	0
UBK292	0	0	4	0	570	13	0	0	0	0	0	0	587
UBK294	0	0	0	0	0	0	0	0	0	0	0	0	0
UBK295	0	0	0	0	0	0	0	0	0	0	0	0	0
UJC004	0	0	0	0	1	6	6	0	0	0	0	0	13
UJC005	0	0	1	0	0	43	0	0	0	0	0	0	44
